# COVID-19: introduction of a new lifestyle and diet among the Malaysians

**DOI:** 10.1186/s42269-023-00979-1

**Published:** 2023-01-05

**Authors:** Md. Najmus Sayadat Pitol, Ana Shakirah Md. Sapir

**Affiliations:** 1 Mangrove Silviculture Division, Bangladesh Forest Research Institute, Muzgunni, Khulna, 9000 Bangladesh; 2 Department of Finance, Banking, and Insurance, Graduate Business School, Universiti Malaya, Kuala Lumpur, Malaysia

**Keywords:** COVID-19, Difficulties, Lifestyle, Lockdown, Online, Pandemic

## Abstract

**Background:**

The stay-at-home conditions due to the COVID-19 pandemic significantly alter the Malaysian lifestyle, and all Malaysians faced difficulties adopting the new lifestyle. A hypothetico-deductive technique has been conducted in this study, to find out what kind of changes the COVID-19 has brought about in the behavior of Malaysians and how they are coping with the changing lifestyles. According to G* Power 3.1 sample size determination in Malaysia, the entire sample of 112 was sufficient to provide the value for the medium effect size for the computation of the F-tests and the findings were reliable (The Cronbach's alpha values were all above 0.70.). To calculate the mean of the lifestyle during COVID-19, the mean scores range between 1.00 and 5.00 marks indicating much reduced to much increased.

**Results:**

It seemed that the jobs traveling (mean 1.80) and outdoor sports time (mean = 1.94) were somewhat reduced. In contrast, indoor sports activities (mean = 3.01), online games (mean = 2.76), physical exercises (mean = 2.63), and the number of staycations (mean = 2.46) during the pandemic stayed the same. However, religious activities (mean = 3.73), body mass index (mean = 3.54), online shopping (mean = 3.90), sleeping time (mean = 3.43), amount of anxiety (mean = 3.38), amount of caffeine (mean = 3.15), medical consumption (mean = 3.10), watching movies (mean = 3.26), and watching drama series (mean = 3.37) during COVID-19 were somewhat increased. In addition, respondents' time spent on social media (mean = 4.27) and online meetings (mean = 4.37) during COVID-19 were much increased. We found no significant differences in the means of the dependent variables (lifestyle of COVID-19) among all demographic characteristics except age and employment status.

**Conclusions:**

New behavioral changes bring new challenges. Malaysians should need to adopt some precautions to minimize the negative effect of new behavioral changes in post-COVID-19 life. The results will help policymakers to make the right decisions about improving the quality of life after COVID-19.

**Supplementary Information:**

The online version contains supplementary material available at 10.1186/s42269-023-00979-1.

## Background

An unexpected and transmissible pandemic coronavirus disease 2019 (COVID-19) outburst in Wuhan, China, in December 2019 (Wang et al. 2020). The world is suddenly stunned and recognizes COVID-19 as a worldwide public health concern. Coronavirus has spread much faster than scientists thought in almost all countries of the world. As a result, the World Health Organization (WHO) declared the outbreak a pandemic and a global public health emergency on March 11, 2020 (WHO, 2020). The World Health Organization has made various restraint plans such as closure of Government, non-government, and educational institutions, markets, amusement parks, and tourist spots, prohibition of large gatherings, ban on all forms of travel and complete lockdowns to avoid viral transmission for all the countries of the world (Ahmed et al 2020; Pedersen and Meneghini 2020; Patwary et al. 2021). Even after so much, many infected cases (about 602 million, with 6.49 million deaths) have been recorded (Our World, 2022). The unpredicted and life-threatening global pandemic has also severely affected Malaysia where about 4.78 million out of 33 million people confirmed cases with 36,210 deaths from January 25, 2020, to September 1, 2022 (Our World, 2022). A large religious gathering of 16,000 Muslims near the capital Kuala Lumpur was responsible for the rapid dispersion of COVID-19 cases. Also, another religious meeting held by Christians in Kuching, Sarawak was accountable for the swift spreading of coronavirus in Malaysia (Tan et al. 2022a, b). Muslims are the majority in Peninsular Malaysia, while Christians lead the non-Malay community in Sabah and Sarawak, and others are Buddhists, Hindus, and Atheists in the Malaysia (Department of Statistics, 2020). Under the Prevention and Control of Infectious Diseases Act 1988, the Malaysian Government implemented the Movement Control Order (MCO) on March 18, 2020, to curb the spread of COVID-19. The MCO limited the movement of people by confining recreational, social, sports, cultural, and religious get-togethers. Traveling was banned nationwide, as also international leaving. The Government was forced to shut down the trades, industries, and government and private educational institutions. Considering the massive loss of livelihoods as all the institutions were closed, the Government introduced a Conditional Movement Control Order (CMCO) instead of an MCO on May 4, 2020. In CMCO, limited exits were allowed for emergency work, and an approval letter from employing organizations was mandatory for employees. The Government also allowed private transport with the condition that not more than four members living under the same roof could be taken as passengers. Public transport services were also permitted with half of the passengers. Besides, taxi cabs, e-cabs, rental cars, etc., run with two passengers (Yong and Sia 2021). No more than 2 people can be seated at the table for dining. After that, the Government replaced the CMCO with Recovery MCO (RMCO) on June 10, 2020, which included work-related travel within Malaysia. It permitted the visit and house gatherings for social and religious festivities (Eid Al-Fitri, Pesta Kaamatan, Gawai Dayak Day, etc.). Any violation of MCO, CMCO, and RMCO's protocols would be punishable with a fine of up to RM1,000 or custody for up to 6 months, or both (Yong and Sia 2021).

All Malaysians faced difficulties adopting the new lifestyle, handling the virus transmission, and worrying about family and friends. The stay-at-home condition was harmful to physical and mental health, increasing the chance of heart disease, stroke, type 2 diabetes, obesity, and so on. The fear of COVID-19, such as sudden isolation, the anxiety of death, losing loved ones, and starvation, has fragile the mental and physical health, as well as the social and economic life of people (Pitol et al. 2022; Rajkumar 2020; Shammi et al. 2020; UN, 2020). Working from home, temporary unemployment, homeschooling children, and a lack of physical touch with other family members, relatives, and peers are all new realities that require time to adjust to (Abdull Rahman et al. 2021, 2022; Hossain et al. 2021; Pitol et al. 2022; Patwary et al. 2020, 2021, 2022 a b, c; Yong and Sia 2021). However, it seemed that the mental and physical health condition varied for various demographic (age, family type, education level, marital status, occupation, etc.) and socioeconomic (income, religion, communication, and transport system, etc.) factors (Marzo et al. 2021, Sekaran and Bougie, 2016). It was found that depression and anxiety disorders were more common for unmarried than married people while females than males, students than services holders, and city people than rural (Marzo et al. 2021).

Moreover, the stay-at-home condition made people obese (30%) and altered their food habits (41%) (Chin et al. 2022; Tan et al. 2022a, b). People uptook more caffeine and consumed more medicine during MCO, CMCO, and RMCO. The Malaysian Chinese males under 24 living on varsity campus consumed more sugar-sweetened caffeine beverages (59.14 g ± 51.28 g per day, 12 teaspoons of sugar) (Chang and Lau 2022) while Malaysian women (92.5%) prepared coffee at home instead of walking out to buy coffee in the market (Ali and Ramanathan 2021), which increased their weight during MCO, CMCO, and RMCO. The movement's cessation carried the economic shutdown in Malaysia. Between January 11 and March 16, 2020, about 170,000 hotel reservations were canceled (Foo et al. 2020), impacting the number of Malaysians who travel and take staycations. The cessation negatively impacts typical forms of exercise and sports activities, primarily the young adults (42.6%) affect the most (Harris et al. 2022). However, the restriction on movement also changed people's relaxation and daily activities. People began to read and listen to Internet news regarding COVID-19 and used phone and online channels to contact family and friends. People were forced to perform their prayers at home, while many worship mansions worldwide have abandoned traditional physical worship and prayers (Tan et al. 2022a, b). The playing time of video and online games also increased. Because of the closure of all cinemas, people were forced to watch movies and dramas online via Disney Plus, HBO Max, and Netflix (Rahman and Arif 2021). He reported that the minimum watching time of drama series and movies was 70 h each month, while the majority showed the “one more episode” syndrome during the stay-at-home condition. Moreover, buying various household chores and groceries online was a fashion during this period. Especially young people of 18–34 years were more likely to purchase products online, and most people buy their products from Shopee (the largest e-commerce site in Malaysia) (Suhardi 2022).

**Fig. 1:**
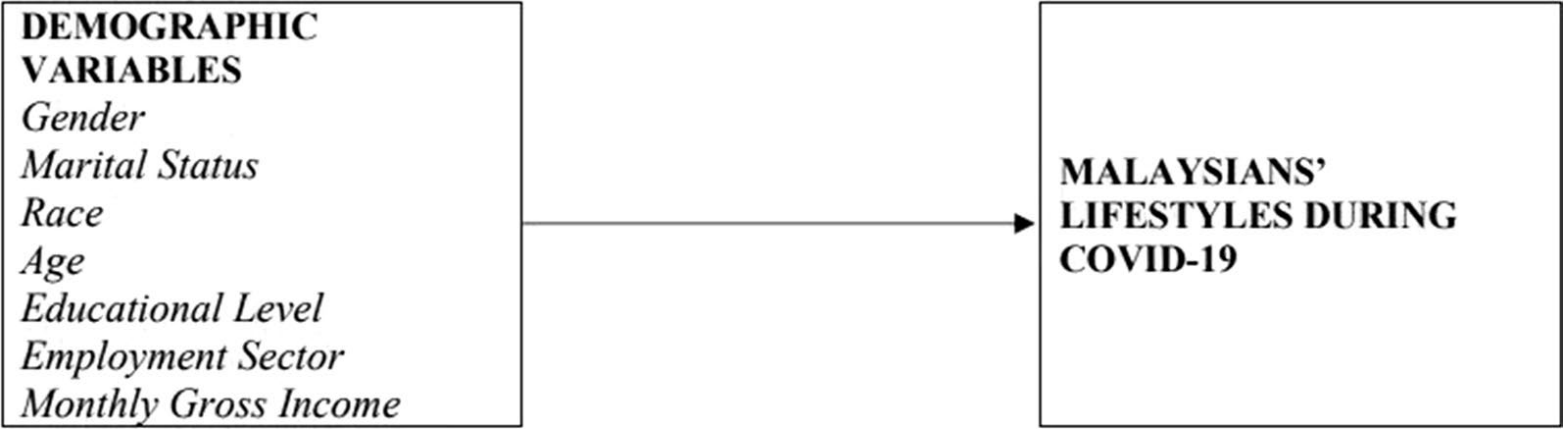
Conceptual framework

Considering all aspects of COVID-19, this study particularly examined the lifestyles among Malaysians, as explained by their sociocultural variables. We perform analysis of variance (ANOVA) and post hoc appraisals to reach our objectives. We hypothesized that the lifestyles during COVID-19 were significantly distinct among gender, marital status, race, age, educational level, employment sector, or monthly gross income. We also assumed that the findings of our study also revealed which demographic variables of Malaysians were disproportionately stricken by COVID-19 and may need to alter their habits drastically. Based on this relationship, the determinants of Malaysians' lifestyles during COVID-19 can be more completely identified in the framework presented in Fig. 1.

## Methods

### Data and sample

A hypothetico-deductive technique allowed the exploratory research to be employed using a cross-sectional study over 3 months. A non-probability convenience sampling technique was employed for data collection. This method was considered the quickest, the most inexpensive sampling design for a large population, and the most relevant, especially during the COVID-19 pandemic. For the sample selection, we targeted working-age Malaysians as the respondents for this study based on the definition of the working-age population provided by the Department of Statistics Malaysia (2020). Therefore, working-age Malaysians, either in or out of the labor force, between 15 and 64, were selected. As the sampling unit consisted of respondents still under parenting control, Malaysians under 18 in 2022 were excluded.

According to the Department of Statistics Malaysia (2020), the total population of Malaysians was 32.4 million, and the working-age population from age ranged between 18 and 64, was estimated to be 22.31 million (or 68.85%). While the number of working-age Malaysians within the labor force was 15.67 million (or 70%), the Department of Statistics Malaysia (2020) also reported that the remaining 6.64 million (or 30%) were outside the labor force (i.e., homemakers, schooling, retired, disabled). Due to broader accessibility to reach as many targeted respondents, this survey was electronically circulated to potential respondents through a snowballing technique to other respondents' acquaintances. We determined the sample size using G* Power 3.1, suggesting that a minimum sample size of 89 was sufficient to provide the value for the medium effect size for the F-test computation.

Before taking the online questionnaire, every Malaysian who volunteered to participate had to disclose their citizenship status through their electronic device (i.e., smartphone) since we provided the survey via Google Form. The respondents were assured that all information gathered would be utilized only for research purposes. Having ascertained the survey participants' eligibility, we invited them to respond to the rest of the questions. Most Malaysians are English oriented, so the questions used English as a communication medium. The structured questionnaire first asked whether they were a Malaysian citizen to establish the respondent's selection criteria. The main content of the questionnaire was divided into two sections: Section A covered the respondents' demographic information, and section B covered the dependent variable of this study, lifestyle during COVID-19.

A total of 133 Malaysian adult respondents responded voluntarily nationwide. Of the 133 submitted questionnaires, 112 (or 84%) were submitted, completed, and answered. Since the total of 112 was above the minimum sample size recommended by G* Power 3.1, this study considered the total of 112 responses appropriate for pursuing analysis. Hoyle (1995) supported that statistical analysis can be conducted with a minimum sample size of 100.

### Instrument and measurement

#### The lifestyle during COVID-19

The lifestyle during COVID-19 is a variable of fundamental interest to us. Eighteen questions about Malaysians' lifestyle during COVID-19; the number of jobs traveling and staycations, body mass index (BMI), anxiety, the amount of sleeping time, the amount of time spent on outdoor indoor sports activities, physical exercises, religious activities, movies, drama series, social media, online shopping, meetings, and virtual games, the amounts of caffeine, fast food, and medical consumptions. They were all measured by using the five-semantic-differential- scale “Much Reduced (1) to Much Increased (5).” The scale rate in the middle represents “Stayed the Same (3).” Since COVID-19 has hugely affected the respondents' daily routine, the insertion of a midpoint on the 5-scale allows survey respondents to express a “Stayed the same” response between much reduced on one side and much increase on the other.

To determine the overall changes in Malaysians' lifestyle during COVID-19, the respondents' feedback for “Much Increased” would be rewarded five marks, “Somewhat Increased” would be awarded four marks, “Stayed the same” would be rewarded three marks, “Somewhat Reduced” would be awarded two marks, and “Much Reduced” would be awarded one mark. To calculate the mean of the lifestyle during COVID-19, the mean scores range between 1.00 and 1.99 marks indicating that COVID-19 has much reduced the routine activities of Malaysians. The score between 2.00 and 2.99 said somewhat reduced. Meanwhile, the midpoint score of 3.00 stayed the same, between 3.01 and 4.00 showed a somewhat increase, and 4.01 – 5.00 indicated a much increase. Mean results were required to employ the parametric statistical techniques in this study.

#### Demographic variables

The demographic variables are the independent variables of this study. Demographic variables were respondents' gender (1 = Male, 2 = Female), marital status (1 = single, 2 = married, 3 = divorced) and race (1 = Malay, 2 = Non-Malay Bumiputera, 3 = Chinese, 4 = Indian), age (1 = 19–24 years old, 2 = 25–30 years old, 3 = 31–36 years old, 4 = 37–42 years old, 5 = 43–48 years old, 6 = 49–54 years old, and 7 = 55–60 years old), educational level (1 = secondary school, 2 = certificate level, 3 = diploma, 4 = professional qualification, 5 = bachelor's degree, 6 = masters' degree, 7 = doctoral degree), employment sector ( 1 = government, 2 = private, 3 = statutory bodies, 4 = self-employed, 5 = unemployed, 6 = students, 7 = retired) and monthly gross incomes (1 = less than RM 2500, 2 = RM 2500–RM 3169, 3 = 3970–RM 4849, 4 = 4850–RM 5879, 5 = RM 5880–RM 7099, 6 = RM 7110–RM 8699, 7 = RM 8700–RM 10,959, 8 = RM 10,690–RM 15,039, 9 = More than RM 15,039). We employed categorical and ordinal scales for measuring demographic variables. Precisely, gender, marital status, race, and employment sector were assessed by using a categorical scale, while an ordinal scale measured age, educational level, and monthly gross income.

### Statistical analysis

Frequencies were performed to present descriptive characteristics. This study's demographics were presented as numbers (N) and frequencies (%), and it informed how many Malaysians gave each response. Frequencies were also performed to describe “The lifestyle during COVID-19.” In the reliability tests, Cronbach's Alpha of 0.70 is recommended as a high degree of internal consistency for indicating the homogeneity of the items in the measures that tap the dependent variables' construct. All items should be capable of independently measuring the same concept so that the respondents attach the same overall meaning to each item. Our demographic variables were much skewed. Therefore, most demographic variables were collapsed for performing ANOVA and other statistical analyses. Instead of removing the respondents from the sample, we recorded them by combining them with the other category. ANOVA was later carried out to understand whether the mean of the dependent variable was significantly distinct among the demographic characteristics, namely, age, educational level, employment sector, and monthly gross incomes. The Tukey’s HSD post hoc further verified the ANOVA results. All statistical analysis was conducted by using SPSS statistical program.

## Results

### Descriptive characteristic

The results revealed that more than 65% of the 112 Malaysian respondents were females, and the remaining 31.3% were males where 50.9% (*n* = 57) of the respondents were married, followed by the single at 46.4% (*n* = 52), and 2.7% (*n* = 3) were divorced (Table 1). The majority of the respondents were Malays (88.4%, *n* = 99), while 8.9% (*n* = 10) were non-Malays' Bumiputera, 1.8% (*n* = 2) were Indians, and 0.9% (*n* = 1) were Chinese in race. In terms of the age groups, 22.3% (*n* = 25) were aged 37–42 years old, followed by the 19–24 years at 20.5% (*n* = 23), 31–36 age group at 19.6% (*n* = 22), and 25–30 at 17.0% (*n* = 19), respectively (Table 1). Another 10.7% (*n* = 12) were aged 43–48, 6.3% (*n* = 7) were aged 49–54, and the remaining 3.6% (*n* = 4) were aged 55–60 years old. As for the level of education, 38.4% (*n* = 43) were master's holders, 32.1% (*n* = 36) were bachelor's degrees, and 12.5% (*n* = 14) were doctoral degrees. The remaining 17% were diploma holders (9.8%, *n* = 11), certificate level (4.5%, *n* = 5), secondary school leavers (1.8%, *n* = 2), and professional qualification (0.9%, *n* = 1), respectively (Table 1). Regarding employment status, most respondents were students (35.7%, *n* = 40), while 32.1% served in the Government of Malaysia, and 21.4% served in the private sector. The remaining 8% were identified as self-employed (5.4%, *n* = 6), unemployed (2.7%, *n* = 3), retired (1.8%, *n* = 2), and served in the statutory bodies (0.9%, *n* = 1). In terms of monthly gross incomes level, 40.2% (*n* = 45) earned monthly income less than RM 2500, 14.3% (*n* = 16) earned RM 2500 to RM 3169 per month, 13.4% (*n* = 15) earned gross revenues ranging between RM 5880–RM 7099 per month, followed by 9.8% (*n* = 11) earned RM 8700–RM 10,959 per month, 5.4% (*n* = 6) RM 4850–RM 5879 per month, 5.4% (*n* = 6) RM 7110–RM 8699 per month), 3.6% (*n* = 4) RM 3170–RM 3969 per month, 3.6% (*n* = 4) RM 3970–RM 4849 per month, 3.6% (*n* = 4) RM 10,690–RM 15,039 per month and 0.9% (*n* = 1) more than RM 15,039 per month (Table 1).

**Table 1:** Descriptive statistics

Variables	Frequency	Percent	Variables	Frequency	Percent
	*n* = 112	(%)		*n* = 112	(%)
**Gender**			**Employment Sector**		
Males	35	31.3	Government	36	32.1
Females	77	68.8	Private	24	21.4
			Statutory Bodies	1	0.9
**Marital Status**			Self-Employed	6	5.4
Single	52	46.4	Unemployed	3	2.7
Married	57	50.9	Students	40	35.7
Divorced	3	2.7	Retired	2	1.8
**Races**			**Monthly Gross Incomes**		
Malays	99	88.4	Less than RM 2,500	45	40.2
Non-Malays’ Bumiputera	10	8.9	RM 2,500 – RM 3,169	16	14.3
Chinese	1	0.9	RM 3,170 – RM 3,969	4	3.6
Indians	2	1.8	RM 3,970 – RM 4,849	4	3.6
			RM 4,850 -RM 5,879	6	5.4
**Age**			RM 5,880 – RM 7,099	15	13.4
19–24	23	20.5	RM 7,110 – RM 8,699	6	5.4
25–30	19	17.0	RM 8,700 – RM 10,959	11	9.8
31–36	22	19.6	RM 10,690 – RM 15,039	4	3.6
37–42	25	22.3	More than RM 15,039	1	0.9
43–48	12	10.7			
49–54	7	6.3			
55–60	4	3.6			
**Educational Level**					
Secondary school	2	1.8			
Certificate Level	5	4.5			
Diploma	11	9.8			
Professional Qualification	1	0.9			
Bachelor's Degree	36	32.1			
Masters' Degree	43	38.4			
Doctoral Degree	14	12.5			

RM 1 = USD 0.20

### Reliability test

We assumed that the result was reliable with the sample in our study. The Cronbach's alpha values were all above 0.70 (see Additional file 1: Table S1). The column “Cronbach's Alpha if Item Deleted” showed the impact of eliminating each item from the scale, which also showed all above 0.70 but below the final alpha obtained.

### Frequencies

#### Frequencies of lifestyles during COVID-19

Based on the result in Table 2, approximately 51.8% of 112 respondents said that their number of jobs traveling (mean = 1.80) during COVID-19 was much reduced. About 52.6% of 112 respondents reported that the number of staycations was somewhat reduced (mean = 2.46), followed by 49.1% of respondents who reported that the body mass index (mean = 3.54) was somewhat increased. We also discovered that roughly half of the 112 respondents thought sleeping time had slightly increased (mean = 3.43). Meanwhile, 38.4% of 112 respondents thought there was a significant reduction in time spent on outdoor sports during COVID-19 (mean = 1.94). In contrast, approximately 36% of 112 respondents said that their amount of time spent on indoor sports activities somewhat increased (mean = 3.01). Similarly, 50% of 112 respondents explained that their physical exercises (mean = 2.63) were somewhat reduced. About 61% of 112 respondents agreed that religious activities somewhat increased during the pandemic (mean = 3.73). Almost 50% of the 112 respondents agreed that anxiety was somewhat increased (mean = 3.38).

**Table 2:** Frequencies of lifestyles during COVID-19

	1	2	3	4	5	Mean	Explanation
	Much reduced	Somewhat reduced	Stayed the same	Somewhat increased	Much increased		
The number of jobs traveling during COVID-19?	58 (51.8%)	27 (24.1%)	18 (16.1%)	9 (8.0%)	0 (0.0%)	1.80	Much reduced
The number of staycations during COVID-19?	37 (33.0%)	22 (19.6%)	28 (25.0%)	15 (13.4%)	10 (8.9%)	2.46	Somewhat reduced
Body mass index (BMI) during COVID-19?	7 (6.3%)	8 (7.1%)	42 (37.5%)	27 (24.1%)	28 (25.0%)	3.54	Somewhat increased
The amount of sleeping time during COVID-19?	7 (6.3%)	11 (9.8%)	38 (33.9%)	39 (34.8%)	17 (15.2%)	3.43	Somewhat increased
The amount of time spent on outdoor sports activities during COVID-19?	43 (38.4%)	41 (36.6%)	21 (18.8%)	6 (5.4%)	1 (0.9%)	1.94	Much reduced
The amount of time spent on indoor sports activities during COVID-19?	17 (15.2%)	19 (17.0%)	36 (32.1%)	26 (23.2%)	14 (12.5%)	3.01	Somewhat increased
The amount of time spent on physical exercises during COVID-19?	17 (15.2%)	39 (34.8%)	31 (27.7%)	19 (17.0%)	6 (5.4%)	2.63	Somewhat reduced
The amount of time spent on religious activities during COVID-19?	4 (3.6%)	11 (9.8%)	29 (25.9%)	35 (31.3%)	33 (29.5%)	3.73	Somewhat increased
The amount of anxiety during COVID-19?	10 (8.9%)	14 (12.5%)	36 (32.1%)	27 (24.1%)	25 (22.3%)	3.38	Somewhat increased
The amount of caffeine consumption during COVID-19?	18 (16.1%)	11 (9.8%)	38 (33.9%)	26 (23.2%)	19 (17.0%)	3.15	Somewhat increased
The amount of fast food consumption during COVID-19?	13 (11.6%)	15 (13.4%)	32 (28.6%)	34 (30.4%)	18 (16.1%)	3.26	Somewhat increased
The amount of medical consumption during COVID-19?	14 (12.5%)	14 (12.5%)	44 (39.3%)	27 (24.1%)	13 (11.6%)	3.10	Somewhat increased
The amount of time for movies during COVID-19?	17 (15.2%)	15 (13.4%)	28 (25.0%)	26 (23.2%)	26 (23.2%)	3.26	Somewhat increased
The amount of time for drama series during COVID-19?	14 (12.5%)	17 (15.2%)	27 (24.1%)	22 (19.6%)	32 (28.6%)	3.37	Somewhat increased
The amount of time spent on social media during COVID-19?	0 (0.0%)	4 (3.6%)	17 (15.2%)	36 (32.1%)	55 (49.1%)	4.27	Much increased
The amount of time for online shopping during COVID-19?	3 (2.7%)	10 (8.9%)	21 (18.8%)	39 (34.8%)	39 (34.8%)	3.90	Somewhat increased
The amount of time online meetings during COVID-19?	3 (2.7%)	3 (2.7%)	10 (8.9%)	30 (26.8%)	66 (58.9%)	4.37	Much increased
The amount of time spent on online games during COVID-19?	38 (33.9%)	8 (7.1%)	28 (25.0%)	19 (17.0%)	19 (17.0%)	2.76	Somewhat reduced

An estimated 50% of the 112 respondents explained that caffeine consumption during COVID-19 was also somewhat increased (mean = 3.15). Almost 50% of 112 respondents said fast food consumption during COVID-19 increased somewhat. 36% of 112 respondents reported that medical consumption during COVID-19 increased somewhat. Around 46% of 112 respondents responded that the time for movies during COVID-19 was somewhat increased (mean = 3.26). Meanwhile, nearly half of the 112 respondents agreed that the time for drama series was increased during COVID-19 (mean = 3.37). More than 80% of the 112 respondents mentioned that the amount spent on social media during COVID-19 was much higher (mean = 4.27). Approximately 70% of 112 respondents agreed that the amount of time for online shopping during COVID-19 was somewhat increased (mean = 3.90). Similarly, nearly 86% of 112 respondents agreed that time spent on online meetings increased significantly during COVID-19 (mean = 4.37). In contrast, 41% of 112 respondents responded that the time spent on online (mean 2.76) games during COVID-19 was somewhat reduced.

### ANOVA with post hoc comparisons

All the demographic variables were collapsed into fewer categories, except gender (see Additional file 1: Table S2), for one-way ANOVA computation. ANOVA was performed to test whether the mean of the dependent variable (lifestyles during COVID-19) was distinct significantly across the demographic variables, namely, age, educational level, employment status, and monthly gross incomes. Based on our ANOVA computation, we discovered no significant differences in the means of the dependent variables (lifestyle of COVID-19) among all demographic characteristics except age and employment status. However, significant lifestyle differences were found during COVID-19 across different generations (Table 3). The youngest respondents have the highest mean of lifestyles during COVID-19 at 0.6812, followed by 25–42 years old, and the older respondents aged 43 and above have the lowest mean at 0.5986. The ANOVA test results do not show which groups differ from one another. Therefore, we further investigated F-Test by reading it in the Tukey’s HSD post hoc. The F-test in post hoc plays a crucial role in detecting a significant difference between some of the tested groups. In this study, Tukey’s HSD post hoc, an integral part of ANOVA, further verified the results. There was a positive direction and significant means at the 0.01 level between (see Table 4):

- Respondents aged 24 and below and 25–42.
- Respondents aged 24 and below and 43 and above.

**Table 3:** ANOVA for age and COVID-19 lifestyles

	*n*	Mean	SD	SE	95% Confidence Interval for Mean		Min	Max
					Lower Bound	Upper Bound		
24 and below	42	0.6812	0.10303	0.01590	0.6491	0.7133	0.42	0.92
25–42	54	0.6144	0.11396	0.01551	0.5833	0.6455	0.30	0.88
43 and above	16	0.5986	0.06413	0.01603	0.5644	0.6328	0.50	0.73
Total	112	0.6372	0.10899	0.01030	0.6168	0.6576	0.30	0.92

Levene's test significant level is 0.095, and homogeneity of variances is assumed. F = 6.128, p < 0.10

**Table 4:** Tukey’s HSD post hoc test about lifestyles of COVID-19 score according to respondents' age

(I) Age	(J) Age	Mean difference (I-J)	Std. Error	Sig	95% Confidence Interval	
					Lower bound	Upper bound
24 and below	25–42	0.06681^***^	0.02145	0.007	0.0158	0.1178
	43 and above	0.08261^**^	0.03063	0.022	0.0098	0.1554
25–42	24 and below	-0.06681^***^	0.02145	0.007	-0.1178	-0.0158
	43 and above	0.01579	0.02968	0.856	-0.0547	0.0863
43 and above	24 and below	-0.08261^**^	0.03063	0.022	-0.1554	-0.0098
	25–42	-0.01579	0.02968	0.856	-0.0863	0.0547

^***^The mean difference is significant at the 0.01 level, ** The mean difference is significant at the 0.05 level, * The mean difference is significant at the 0.10 level

These findings imply that the COVID19 pandemic has significantly changed the lifestyles of respondents aged 24 and below compared to their older counterparts. Moreover, regarding the employment status category, a significant result of 0.10 was found between employment status and lifestyles during COVID-19 (Table 5). The highest mean of lifestyles during COVID-19 was unemployed respondents, students, and retirees (mean 0.6372), followed by private and self-employed (mean 0.6204). The employees of government servants and statutory bodies have the lowest mean at 0.6144. Based on the Tukey’s HSD post hoc test (Table 6), the evidence shows that:

- The unemployed, students, and retiree group have a significant mean difference at the 0.10 level with Government & Statutory Bodies employees.

**Table 5:** ANOVA for employment sector and COVID-19 lifestyles

	*n*	Mean	SD	SE	95% Confidence Interval for Mean	Min	Max	
					Lower Bound	Upper Bound		
Government & Statutory Bodies	37	0.6144	0.11638	0.01913	0.5756	0.6532	.30	.83
Private and Self-Employed	30	0.6204	0.09177	0.01675	0.5861	0.6546	.46	.80
Unemployed, Students & Retired	45	0.6672	0.10856	0.01618	0.6345	0.6998	.37	.92
Total	112	0.6372	0.10899	0.01030	0.6168	0.6576	.30	.92

Levene's test significant level is 0.730, and homogeneity of variances is assumed. F = 2.968, p < 0.10

**Table 6:** Tukey’s HSD post hoc test about lifestyles of COVID-19 score according to respondents' employment

(I) Employment	(J) Employment	Mean difference (I–J)	S.E	Sig	95% confidence interval	
					Lower bound	Upper bound
Government & Statutory Bodies	Private and Self-Employed	-0.00596	0.02631	0.972	-0.0685	0.0566
	Unemployed, Students & Retired	-0.05275*	0.02377	0.072	-0.1092	0.0037
Private and Self-Employed	Government & Statutory Bodies	0.00596	0.02631	0.972	-0.0566	0.0685
	Unemployed, Students & Retired	-0.04679	0.02524	0.157	-0.1068	0.0132
Unemployed, Students & Retired	Government & Statutory Bodies	0.05275*	0.02377	0.072	-0.0037	0.1092
	Private and Self-Employed	0.04679	0.02524	0.157	-0.0132	0.1068

^***^The mean difference is significant at the 0.01 level, ** The mean difference is significant at the 0.05 level, * The mean difference is significant at the 0.10 level

Overall, we can conclude that the COVID-19 pandemic has significantly changed the lifestyles of the respondents outside the labor force (i.e., unemployed, students, and retiree) rather than those who participate in the labor force (i.e., Government & Statutory Bodies)

## Discussion

COVID-19 affects our physical and mental health and social and economic parts. This study explores the determinant factors of Malaysians, namely, gender, marital status, race, age, educational level, employment sector, and monthly gross incomes on their lifestyles during COVID-19. The questionnaires were conveniently distributed among Malaysian adults aged 18 years old at the point of the survey nationwide, with 112 respondents for our analysis. The one-way ANOVA test was conducted for the demographic variables: age, educational level, employment status, and monthly gross income. This means the significant relationship between demographic factors and lifestyles of COVID-19 explains which generation cohort was primarily affected by the Government's restriction during the pandemic, as well as continuing to put up with the new lifestyles until now. Gender and marital status were excluded from the ANOVA test because they only have two different groups. Among the demographic factors tested, ANOVA can only detect two determinant factors that primarily change lifestyles: age and employment. In this research, respondents' age was measured using an ordinal scale, while the employment sector was estimated using a categorical scale. The five-semantic-differential measured the lifestyles during COVID-19; respondents were asked to indicate their activities by using bipolar adjectives of much reduced–much increased.

In a nutshell, the empirical evidence of Tukey’s post hoc supports the view that the COVID-19 pandemic in Malaysia primarily affects the lifestyles of young adults, the unemployed, and students. This result suggests that COVID-19 essentially changed the lifestyles of 26% of Malaysia's 33 million population (8.5 million). The youngest group in this study represents Malaysians born between 1997 and 2010, which we call Generation Z. In Malaysia, the Generation Z cohort tends toward music and arts more than the older generation, who are more concentrated in the corporate field (Mothersbaugh & Hawkins 2016). Nik Jaafar et al. (2021) discovered that Malaysia's young adults were born during the digital era, relying extensively on the Internet to do nearly all of their chores. Many Generation Z is college students, fresh graduates, or at the beginning of their career before COVID-19 hit, making them connected with insufficient income. The most alarming thing was that Generation –Z was addicted to the virtual world, like online games (PUBG., free fire, Clash of Clans, etc.), movies, dramas, Facebook, YouTube, and so on (Pitol et al. 2022). The condition was more severe during the COVID-19 lockdown due to academic delay (Agnew et al. 2019) and uncertain careers (Sahu 2020) for Generation Z.

However, the professional expeditions were much reduced, and a much decrease followed in time spent participating in outdoor activities. The respondents also said that the amount spent on Internet gaming and physical workouts decreased slightly. Foo et al. (2020) and Harris et al. (2022) explored that the execution of MCO and CMCO caused the revocation of many professional trips and outdoor activities. The introduction of work-from-home lifestyles may reduce the time spent on Internet gaming due to the need to install workstation software on personal computers. Respondents also stated that the number of staycations throughout the epidemic was somewhat reduced. In contrast to the quantity of time spent on indoor sporting activities, were somewhat increased. On the contrary, Pitol et al. (2022) found the opposite result in Bangladesh. Moreover, religious activities, BMI, Internet shopping, sleeping time, anxiety, caffeine consumption, medical consumption, watching movies, and watching drama series increased slightly during COVID-19. This result is somewhat consistent with the findings of previous research such as Chang & Lau (2022), Suhardi (2022), Tan et al. (2022a, b), Ali & Ramanathan (2021), Marzo et al. (2021), and Rahman & Arif (2021). The long-lasting stay-at-home condition caused emotional sickness in the people (Lambert et al. 2002). It has also increased the worried, panicked, and devitalized social networks during any epidemic and emergency (UN 2020; IASC 2020). Respondents' time spent on social media and online meetings, on the other hand, grew significantly during COVID-19. Most professional meetings are held online using Google Meet, Zoom, Telegram, etc., saving time and money.

The way Malaysians' lifestyles were presented was more consistent with News Strait Times (2020), which listed the unique ways Malaysians spend time during the lockdown period. Malaysians also took the opportunity of MCO to transform mundane apartments into beautiful homes. They also tested their cooking skills by trying out new recipes for lunch and dinner. They also participated in whipping the coffee's Internet challenge, yoga, reading books, and home gardening, instead of spending much time indoors in sports, online games, and staycations. Many people have resorted to the Internet to upload images and video footage of the transformation process of the house. New recipes and coffee whipping have been extensively circulated, sparking the inspiration to fellow Malaysians on the Internet to see if they can also do the same.

Currently, the incidence of the COVID-19 has reduced a lot, and life is getting normal. But the long-standing COVID-19 has changed many habits of our daily life. COVID-19 has also changed many of our eating habits, including hot water uptake, more consumption of coffee and tea, etc. However, COVID-19 has introduced virtual classrooms, online meetings and shopping, and working from home, an essential part of our daily lives. Therefore, the Government must take conscious steps to improve the quality of life after COVID-19. We hope that our results will be beneficial in that regard. Moreover, this study gives an idea (medium size effect) about the lifestyle during COVID-19 and give guideline to us on what should we do during the epidemic condition. More extensive research will be needed including all the races and ethnic groups, occupations, children, worker classes, etc. to attain a specific idea about the effect of the COVID-19.

## Conclusions

The COVID-19 pandemic has significantly changed the lifestyles of respondents aged 24 and below compared to their older counterparts. It also changed the lifestyles of the unemployed, students, and retiree person rather than those who work in Government & Statutory Bodies. This information plays a vital role in the policymakers for implementing the new policy that improves the post-COVID-19 life of Malaysians. We should show special care to young adults, the unemployed, students, and retirees during and after the pandemic condition in the future. However, we should try our best to maintain our normal daily activities (as possible) that minimize the negative effect on our mental and physical health during the epidemic. We will maintain communication with our family and friends via online platforms for making ourselves tension free.

## Supplementary Information

**Additional file 1. Table S1**. Reliability Test. **Table S2.** Collapsing the number of categories of the variables.

## Data Availability

The raw data are available from the authors upon reasonable req

## Abbreviations

WHO: World Health Organization
MCO: Movement Control Order
CMCO: Conditional Movement Control Order
RMCO: Recovery Movement Control Order
BMI: Body mass index
RM: Malaysian Ringgit
Generation Z: The generation reaching adulthood in the second decade of the twenty-first century, perceived as being familiar with the Internet from a very young age
