# Supplementary material for: COVID-19: introduction of a new lifestyle and diet among the Malaysians
Source: Bull Natl Res Cent. 2023 Jan 5;47(1):3. doi: 10.1186/s42269-023-00979-1 (PMC9815062; doi:10.1186/s42269-023-00979-1)
Supplement: Supplementary file 1 — Additional file 1. Table S1. Reliability Test. Table S2. Collapsing the number of categories of the variables. [file 42269_2023_979_MOESM1_ESM.docx]

**Supplementary material**

Supplementary Table 1 (S1) showed the reliability tests of our sample. We found that the result was reliable with the sample in our study. In the reliability tests, Cronbach's Alpha of 0.70 was recommended as a high degree of internal consistency for indicating the homogeneity of the items in the measures that tap the dependent variables' construct.

Supplementary Table 2 (S2) shows the frequency and percentage of the demographic variables. Our demographic variables were much skewed. Therefore, most demographic variables were collapsed (before and after the collapse) for performing ANOVA and other statistical analyses. Instead of removing the respondents from the sample, we recorded them by combining them with the other category.

| **S1. Reliability Test** | | |
| --- | --- | --- |
| Dimension | Cronbach's Alpha | |
|  | Item If Deleted | Reliability Value |
| *Lifestyles during Covid-19 (18 items)* |  | 0.788 |
| The number of jobs traveling during Covid-19? | 0.780 |  |
| The number of staycations during Covid-19? | 0.785 |  |
| Body mass index (BMI) during Covid-19? | 0.779 |  |
| The amount of sleeping time during Covid-19? | 0.777 |  |
| The amount of time spent on outdoor sports activities during Covid-19? | 0.780 |  |
| The amount of time spent on indoor sports activities during Covid-19? | 0.772 |  |
| The amount of time spent on physical exercises during Covid-19? | 0.786 |  |
| The amount of time spent on religious activities during Covid-19? | 0.786 |  |
| The amount of anxiety during Covid-19? | 0.785 |  |
| The amount of caffeine consumption during Covid-19? | 0.784 |  |
| The amount of fast food consumption during Covid-19? | 0.765 |  |
| The amount of medical consumption during Covid-19? | 0.781 |  |
| The amount of time for movies during Covid-19? | 0.770 |  |
| The amount of time for drama series during Covid-19? | 0.772 |  |
| The amount of time spent on social media during Covid-19? | 0.774 |  |
| The amount of time for online shopping during Covid-19? | 0.780 |  |
| The amount of time online meetings during Covid-19? | 0.782 |  |
| The amount of time spent on online social games during Covid-19? | 0.773 |  |

| **S2. Collapsing the number of categories of the variables** | | | | | | |
| --- | --- | --- | --- | --- | --- | --- |
| Before collapse | | |  | After collapse | | |
| Variables | Frequency | Percent |  | Variables | Frequency | Percent |
|  | *n*=112 | (%) |  |  | *n*=112 | (%) |
| **Gender** |  |  |  | **Gender** |  |  |
| Males | 35 | 31.3 |  | - | - | - |
| Females | 77 | 68.8 |  | - | - | - |
| **Marital Status** |  |  |  | **Marital Status** |  |  |
| Single | 52 | 46.4 |  | Single | 52 | 46.4 |
| Married | 57 | 50.9 |  | Married | 60 | 53.6 |
| Divorced | 3 | 2.7 |  | - |  |  |
| **Races** |  |  |  | **Races** |  |  |
| Malays | 99 | 88.4 |  | Malays | 99 | 88.4 |
| Non-Malays’ Bumiputera | 10 | 8.9 |  | Non-Malays | 13 | 11.6 |
| Chinese | 1 | 0.9 |  | - | - | - |
| Indians | 2 | 1.8 |  | - | - | - |
| **Age** |  |  |  | **Age** |  |  |
| 19-24 | 23 | 20.5 |  | 24 and below | 42 | 37.5 |
| 25-30 | 19 | 17.0 |  | 25-42 | 54 | 48.2 |
| 31-36 | 22 | 19.6 |  | 43 and above | 16 | 14.3 |
| 37-42 | 25 | 22.3 |  | - | - | - |
| 43-48 | 12 | 10.7 |  | - | - | - |
| 49-54 | 7 | 6.3 |  | - | - | - |
| 55-60 | 4 | 3.6 |  | - | - | - |
| **Educational Level** |  |  |  | **Educational Level** |  |  |
| Secondary school | 2 | 1.8 |  | Diploma and below | 18 | 16.1 |
| Certificate Level | 5 | 4.5 |  | Bachelor's Degree | 37 | 33.0 |
| Diploma | 11 | 9.8 |  | Masters and Ph.D | 57 | 50.9 |
| Professional Qualification | 1 | 0.9 |  | - | - | - |
| Bachelor's Degree | 36 | 32.1 |  | - | - | - |
| Masters' Degree | 43 | 38.4 |  | - | - | - |
| Doctoral Degree | 14 | 12.5 |  | - | - | - |
| **Employment Sector** |  |  |  | **Employment Sector** |  |  |
| Government | 36 | 32.1 |  | Government & Statutory Bodies | 37 | 33.0 |
| Private | 24 | 21.4 |  | Private & Self-Employed | 30 | 26.8 |
| Statutory Bodies | 1 | 0.9 |  | Unemployed, Students & Retired | 45 | 40.2 |
| Self-Employed | 6 | 5.4 |  | - | - | - |
| Unemployed | 3 | 2.7 |  | - | - | - |
| Students | 40 | 35.7 |  | - | - | - |
| Retired | 2 | 1.8 |  | - | - | - |
| **Monthly Gross Incomes** |  |  |  | **Monthly Gross Incomes** |  |  |
| Less than RM 2,500 | 45 | 40.2 |  | Less than RM 2,500 | 45 | 40.2 |
| RM 2,500 – RM 3,169 | 16 | 14.3 |  | RM 2,500 – RM 3,169 | 30 | 26.8 |
| RM 3,170 – RM 3,969 | 4 | 3.6 |  | RM 3,170 and above | 37 | 33.0 |
| RM 3,970 – RM 4,849 | 4 | 3.6 |  | - | - | - |
| RM 4,850 -RM 5,879 | 6 | 5.4 |  | - | - | - |
| RM 5,880 – RM 7,099 | 15 | 13.4 |  | - | - | - |
| RM 7,110 – RM 8,699 | 6 | 5.4 |  | - | - | - |
| RM 8,700 – RM 10,959 | 11 | 9.8 |  | - | - | - |
| RM 10,690 – RM 15,039 | 4 | 3.6 |  | - | - | - |
| More than RM 15,039 | 1 | 0.9 |  | - | - | - |
| **Notes :** * *One-Way ANOVA would be applicable for analyzing age, educational level, employment status, and monthly gross incomes* | | | | | | |
